# Analysis of Physiological, Technical, and Tactical Analysis during a Friendly Football Match of Elite U19

**DOI:** 10.3390/sports4020035

**Published:** 2016-06-16

**Authors:** Juan Ignacio Ortega, Carlos Evangelio, Filipe Manuel Clemente, Fernando Manuel Lourenço Martins, Sixto González-Víllora

**Affiliations:** 1 Physical Education Department, University of Castilla-La Mancha, Edificio Fray Luis de León, 16071 Cuenca, Spain; naichzzz@gmail.com (J.I.O); carletes_92@hotmail.com (C.E); 2 Instituto Politécnico de Viana do Castelo, Escola Superior de Desporto e Lazer, Complexo Desportivo e Lazer Comendador Rui Solheiro—Monte de Prado, 4960-320 Melgaço, Portugal; filipe.clemente5@gmail.com; 3 Instituto de Telecomunicações, Delegação da Covilhã, Instituto Superior Técnico—Torre Norte —Piso 10, Av. Rovisco Pais, 1, 1049-001 Lisboa, Portugal; fmlmartins@ubi.pt; 4 Instituto Politécnico de Coimbra, Escola Superior de Educação, Departamento de Educação, IIA, RoboCorp, ASSERT, Rua Dom João III, 3030-329 Coimbra, Portugal

**Keywords:** performance analysis, match analysis, network analysis, soccer, adjacency matrices

## Abstract

The main objective was to analyze a friendly match of youth elite soccer players identifying the variance of tactical and physiological response parameters during the game. In addition, detecting the impact of both halves on player performance. For the purposes of this study twenty-two U19 players were analyzed playing 11v11. Activity profile, heart rate (HR and HRmax), grouped in five different zones were analyzed via Bluetooth technology, technical performance was analyzed by the Team Sport Assessment Procedure (TSAP), and tactical performance was measured by Social Network Analysis. A comparison of heart rate responses showed significant main effects in the halves (*p* = 0.001; $\eta_{p}^{2}$ = 0.623). A comparison between tactical position and technical performance had significant main effects (*p* = 0.001; $\eta_{p}^{2}$ = 0.390). Tactical position showed statistically significant effects on tactical prominence (*p* = 0.002; $\eta_{p}^{2}$ = 0.296). Therefore, fatigue is a component distinguished in technical/tactical parameters, such as volume of play and efficiency index. Results suggest that fatigue effects may constrain technical performance and, for that reason, the use of instruments to monitor the fatigue effect during matches may be suggested.

## 1. Introduction

The game of football requires a great dynamic and movements that depend on the ball location, teammates, and opponents’ behaviors [1,2]. The movements made by players constrains the physiological and physical demands; thus, team dynamics should be analyzed in association with the personal demands [3]. Despite this, the analysis has been performed not considering the dynamic of the game. Match analysis (about the patterns of play) and performance analysis (based on internal and external load) have been working alone without an association.

Research conducted on soccer have been found that heart rate (HR) responses varies between 80% and 90% of HRmax and ~75% of VO_2max_ [4,5]. These values are in line with the lactate threshold, thus prominently varying between oxidative and glycolytic systems [6]. These acute responses depend from the time-motion profile of the players. Commonly, 10–12 km per game are covered by players during a full match [7]. During the match, 1000 to 1400 short activities changing every 4–6 s are made [8]. Moreover, technical activity and performance may also influence the individual impact of the game in a player [9]. Thus, time-motion analysis and acute physiological responses confirms the variability of demands that occurs during the match.

Nevertheless, the time-motion profile is constrained by the specificity of a team’s dynamics and decisions made during the emergence of the game [10,11]. Some observational methods have characterized the team dynamics by using some mathematical algorithms and observational methods [12,13]. In the specific case of teammates’ interactions, network analysis based on graph theory have been used [14,15]. Results have showed that midfielders and external defenders are the prominent players in the building of passing sequences [16]. On the other hand, during attacking transition and counter-attack, forwards assume a great prominence [17]. Thus, the phases of the game and the tactic specificity of the team contribute to the individual participation of each player during the match.

Despite of this isolated analysis to acute physiological responses and collective organization, no study has analyzed both at the same time, as far we know. However, such analysis is extremely important to identify the impact of the style of play in the physiological demands. This information will help optimize the training methodology and specificity.

For that reason, the aim of this study is to analyze a full match of youth elite soccer players and identify the variance of physiological and collective organization parameters during the game. It also aims to identify the impact of halves on the performance.

## 2. Methods

### 2.1. Participants

Twenty-two elite youth soccer players (age 19.85 ± 1.2 years old; height 178.91 ± 4.5 cm; body mass 71.56 ± 6.1 kg; competitive playing experience 9.23 ± 1.8 years) from two squads of a Spanish National Youth Premier League soccer club (highest state competition) volunteered to participate in this study. Eleven players from each squad were selected to participate in the study and were free of illness and injury at the time of testing. A minimum of five years of experience in elite youth soccer and the regular four weekly training sessions were the criteria to include the players in this study. All participants signed informed consent forms outlining commitment, benefits, potential risks, and the study’s procedures in line with the ethical standards of Declaration of Helsinki for the study in humans. The study also complied with ethical standards of the University of Castilla-la Mancha.

### 2.2. Procedures of Heart Rate Monitoring

An initial session was used to assess fitness levels and familiarize participants with all testing procedures. Participants completed the Yo-Yo Intermittent Recovery Test level 1 by using soccer boots in an official turf field during which HRmax was determined [18]. The HR data was recorded via Bluetooth technology (Polar Team App, Polar Electro Oy, Pakkalankuja, Finland) in all training sessions. The HR results were grouped into five different zones of % HRmax: zone 1 [Z1] (50–60 % HRmax), zone 2 [Z2] (60–70 % HRmax), zone 3 [Z3] (70–80 % HRmax), zone 4 [Z4] (80–90 % HRmax), and zone 5 [Z5] (≥90 % HRmax). After the preliminary testing protocol, the 11v11 game was promoted in an official turf field during two halves of 30 min with 15 min of rest. A smaller period of time (30 min and not the regular 45) was used based on the temperature of 25 °C recorded during the match.

### 2.3. Assesment of Technical Performance

The technical performance it was analyzed following the Team Sport Assessment Procedure (TSAP) protocol [19,20]. This instrument allowed to identify the following observational indicators [20]: (CB) Conquered Ball represents the moment in which the player intercepts the ball from the opponent; (RB) Received Balls represent the moment in which the player receives the ball from a teammate; (LB) Lost Ball represents the action of losing control of the ball; (NB) Neutral Ball represents a routine pass made without penetrating the opponent’s region or does not allow moving forward; (P) Pass that allows displacing the ball into the opponent’s defensive region; and (SS) Successful Shot on Goal, which represents the action of scoring or a shot that ensures to the team keeps possession of the ball. Using the indicators, it was possible to compute the following indices of technical performance: (i) volume of play (Volume of Play (VP)=CB+RB); (ii) attacks with ball (Attacks with ball (AB)=P+SS); (iii) efficiency index ($Efficiency\;Index\;\left(EI\right)=\frac{AB}{10+LB}$); and (iv) performance score ($Performance\;Score\;\left(PS\right)=\left(\frac{VP}{2}\right)+\left(EI\times 10\right)$).

The observational process was made after video collection by the same researcher with experience in match analysis. The reliability was tested by using a test-retest protocol (20-day interval) following by the Cohen’s Kappa test [21]. After testing 15% of the full data, a Kappa value of 0.95 was observed, thus achieving a recommended value for this type of procedure [21].

### 2.4. Network Measurements

In our study it was analyzed the teammates’ interaction during attacking moments based on a Social Network Analysis. This approach have been used in the last few years in order to identify the tendencies of interactions between teammates [22]. In our case only attacking interaction represented by passes between teammates it was analyzed. The protocol of observation followed previous studies in this field of analysis [23]. The following network metrics were computed in the SocNetV (version 1.9).

### 2.5. OutDegree

The OutDegree metric identifies the centrality level of a player in to pass the ball to their teammates. The following algorithm was used for the case of weighted digraphs (directed graphs), as a standard measure [24]; that is, the proportion of weights of the nodes that are adjacent to *n_i_*.

$${C\prime }_{\left(D-out\right)}^{w}\left(n_{i}\right)=\frac{k_{i}^{w-out}}{\sum_{i=1}^{n}\sum_{\begin{array}{c} j=1\\ j\neq i \end{array}}^{n}a_{ij}},$$

### 2.6. InDegree

The InDegree represents the centrality level of a player in the team. Specifically, it identifies the most recruited players to receive the ball. The following algorithm it was used in order to standardize the group size *n* [24]; that is, the proportion of weights of the nodes that are adjacent to *n_i_*.

$${P\prime }_{\left(D-in\right)}^{w}\left(n_{i}\right)=\frac{k_{i}^{w-in}}{\sum_{i=1}^{n}\sum_{\begin{array}{c} j=1\\ j\neq i \end{array}}^{n}a_{ij}},$$

### 2.7. Betweennees Centrality

Betweenness centrality measures the intermediate players between neighbors. The standard of betweenness centrality for weighted digraphs is calculated as [25]:

$${C\prime }_{b}\left(n_{k}\right)=\frac{C_{b}\left(n_{k}\right)}{\left(n-1\right)\left(n-2\right)}$$

### 2.8. Statistical Procedures

The influences of tactical position and halves of match on the average % HRmax, % Time per HRmax Zone (% of total time), volume of play, efficiency index, performance score, % InDegree, % OutDegree, and % Betweenness were analyzed using two-way MANOVA after validating normality and homogeneity assumptions. The assumption of normality for each univariate dependent variable was examined using Kolmogorov-Smirnov tests (*p* > 0.05). The assumption of the homogeneity of each group’s variance/covariance matrix was examined with the Box’s M Test. When the MANOVA detected significant statistical differences between the two factors, we proceeded to the two-way ANOVA for each dependent variable, followed by Tukey’s HSD *post hoc* test [26]. When the two-way ANOVA showed an interaction between factors, it also generated a new variable that crossed the two factors (e.g., GK * 1st half; GK * 2nd half) for each dependent variable to identify statistical significance. Ultimately, the statistical procedures used were one-way ANOVA and Tukey HSD *post hoc*. If no interactions were detected in two-away ANOVA, a one-way ANOVA was used for each independent variable. All statistical analyses were performed using IBM SPSS Statistics (version 22) at a significance level of *p* < 0.05. The following scale was used to classify the effect size (partial eta square) of the test [27]: small, 0.2–0.49; moderate, 0.50–0.79; and large, 0.80–1.

## 3. Results

### 3.1. Heart Rate Analysis

The two-way MANOVA revealed that the halves (*p* = 0.001; $\eta_{p}^{2}$ = 0.623; moderate effect size) had significant main effects on the heart rate responses. No statistical differences were found between tactical positions (*p* = 0.376; $\eta_{p}^{2}$ = 0.160; small effect size). There was no significant interaction (Pillai’s Trace = 0.572; F = 0.946; *p* = 0.541; $\eta_{p}^{2}$ = 0.143; small effect size) between tactical position and halves on heart rate responses.

In the case of % HRmax average, Z1, Z2, Z3, Z4, and Z5, a one-way ANOVA was performed on each independent variable because no interaction was found between factors. The statistical values resulted from the comparison between tactical positions can be observed in the following Table 1.

One-way ANOVA was also carried out to compare the values between formats for the variables of % HRmax average, Z1, Z2, Z3, Z4, and Z5. The values can be verified in the following Table 2.

### 3.2. Technical Performance

The two-way MANOVA revealed that the tactical position (*p* = 0.001; $\eta_{p}^{2}$ = 0.390; small effect size) had significant main effects on the technical performance. No statistical differences were found between halves (*p* = 0.173; $\eta_{p}^{2}$ = 0.107; small effect size). There was no significant interaction (Pillai’s Trace = 0.241; F = 0.878; *p* = 0.558; $\eta_{p}^{2}$ = 0.121; small effect size) between tactical position and halves on technical performance.

In the case of Volume of Play, Efficiency Index, and Performance Score, a one-way ANOVA was performed on each independent variable because no interaction was found between factors. The statistical values resulted from the comparison between tactical positions can be observed in the following Table 3.

The one-way ANOVA it was also carried out to compare the values between formats for the variables of Volume of Play, Efficiency Index, and Performance Score. The values can be verified in the following Table 4.

### 3.3. Network Analysis

The two-way MANOVA revealed that the tactical position (*p* = 0.002; $\eta_{p}^{2}$ = 0.296; small effect size) had significant main effects on the tactical prominence. No statistical differences were found between halves (*p* = 0.678; $\eta_{p}^{2}$ = 0.049; small effect size). There was no significant interaction (Pillai’s Trace = 0.207; F = 0.475; *p* = 0.948; $\eta_{p}^{2}$ = 0.069; small effect size) between tactical position and halves on network variables.

In the case of % InDegree, % OutDegree, and % Betweenness, a one-way ANOVA was performed on each independent variable because no interaction was found between factors. The statistical values resulted from the comparison between tactical positions can be observed in the following Table 5.

One-way ANOVA was also carried out to compare the values between formats for the variables of network density, clustering coefficient, and total arcs. The values can be verified in the following Table 6.

## 4. Discussion

The objective of this study was to analyze the influence of tactical positioning of the players in a friendly football match during two halves of 30 min. Moreover, heart rate response, technical analysis, and collective behavior were related with parameters.

Statistical differences in HR responses were found between halves. Descriptive statistics showed that external defenders, midfielders, and forwards increased HR values from the 1st to the 2nd half. Opposite effects were found in the remaining tactical positions. Generally, HR values, blood lactate concentrations and VO_2_max decreases from the 1st to the 2nd half based on the decrease of carbohydrates and glycogen storage [8,28,29].

Moreover, when the fatigue increases in the 2nd half, the efficiency index decreases for all playing positions, except for the forward, who increases his volume of play to take advantage of the tiredness of the defenders. Thus, forwards covered more distance than defenders [30] when they can create chances of goal for the condition of play.

Consequently, the volume of play decreases for the tactical playing position, in the same way, than that of the efficiency index. This is due to the possession of the ball replacing the attack play in the 2nd half since fatigue does not allow effective vertical play due to reduced physical condition. The coordination, the muscle participation, agility, and the decision-making of the game are affected due to fatigue [4].

Moreover, the style of play decided by the coach has an influence on the volume of play. In this way, the team evaluated has a direct style of play for the attacking phases, and a pressuring of the ball for the defensive phases of the game, attending for the physiological, physical, and technical/tactical demands of the team [31]. For this reason, forwards increase their volume of play and efficiency index.

On the one hand, % InDegree between halves increase in positions without risk of losing the ball, like external defenders or central midfielders. Moreover, the Z5 of the % HR (Table 2) show an increase for external defenders, who participate more in the play of the team [16].

On the other hand, % OutDegree is increased in the 2nd half when fatigue is higher, and implies more mistakes by the players. The external defenders have less left back, while the midfielders have the greatest number of left back because they covered more distance than the rest of the tactical positions, taking more frequency of right back [30]. Therefore, midfielders have more % Betweeness [14] because the direct style of play decreases on the 2nd half.

Finally, the performance score has the same results than that of the volume of play because it is diminished for tactical positions, except for the forward, who gets goals because of the increase in his volume of play and chances for goals. Moreover, with a direct style of play, possession with long passing sequences will decrease and this may increase the chances of a goal [32].

In conclusion, this study constitutes a vision of the tactical/technical demands of the play on players of different tactical positions in relation to the heart rate response and collective behavior. For this reason, the practical applications of this study allow knowing the features of the system of the play depending on each moment of the match. Specifically, in relation with the volume of play, the efficiency index, and performance score, the tactical positions are more relevant in each feature. Furthermore, if we can control physiological parameter demands in soccer, we could analyze more deeply, such as economizing on energy or nutrient reserves. Moreover, other studies could use this investigation to go into detail for a specific tactical position, or to analyze a specific variable of the technical performance and their repercussion on each moment of the match.

On the other hand, this study has limitations in order to know the responses when the players participate in a competition match, where other factors like the concentration or vigor of the players can be altered. Another important limitation was the small sample used in this initial study. Moreover, it is recommended that investigations for other ages be conducted, in order to conclude results for other players’ features. Furthermore, it is necessary the improvement of technology be in agreement with this area of research in order to strive for excellence in the exhaustive analysis of parameters.

## 5. Conclusions

The objective of this study was to analyze the influence of tactical positioning of the players in a friendly football match. Furthermore, different physiological and technical parameters were measured. The results suggest that fatigue is a component distinguished in the volume of play and efficiency index. When fatigue increases, the implication is that more mistakes are made by the players and these parameters decrease. Therefore, the study may suggest the relevance of measuring physiological, technical, and tactical parameters in order to apply a performance training and system knowledge of the game.

## Figures and Tables

**Table 1:** One-way ANOVA values of halves in each tactical position in % HRmax average, Z1, Z2, Z3, Z4, and Z5.

Variable	Half	Position	M(SD)	F	*p*	$\eta_{p}^{2}$
% HRmax average	1st half	GK	–	1.325	0.299	0.228
		ED	69.00 (15.94)			
		CD	82.33 (7.57)			
		CMF	70.63 (17.85)			
		EMF	85.00 (4.90)			
		FW	67.00 (18.52)			
	2nd half	GK	–	1.427	0.265	0.241
		ED	79.75 (5.06)			
		CD	77.00 (3.61)			
		CMF	75.25 (6.90)			
		EMF	74.20 (6.57)			
		FW	69.00 (5.57)			
Z1	1st half	GK	–	0.936	0.465	0.172
		ED	7.50 (9.26)			
		CD	0.33 (0.58)			
		CMF	8.50 (12.87)			
		EMF	0.80 (1.79)			
		FW	9.67 (8.74)			
	2nd half	GK	–	0.706	0.598	0.136
		ED	4.25 (6.65)			
		CD	5.33 (6.11)			
		CMF	12.00 (10.85)			
		EMF	8.60 (7.16)			
		FW	9.00 (4.58)			
Z2	1st half	GK	–	0.385	0.816	0.079
		ED	7.75 (5.68)			
		CD	4.00 (4.58)			
		CMF	6.00 (5.83)			
		EMF	4.40 (3.65)			
		FW	7.33 (5.51)			
	2nd half	GK	–	1.368	0.284	0.233
		ED	14.75 (3.76)			
		CD	19.33 (4.34)			
		CMF	19.25 (2.66)			
		EMF	26.20 (3.36)			
		FW	19.33 (4.34)			
Z3	1st half	GK	–	0.496	0.739	0.099
		ED	19.25 (13.07)			
		CD	35.33 (26.84)			
		CMF	24.50 (17.91)			
		EMF	23.00 (14.83)			
		FW	31.00 (8.72)			
	2nd half	GK	–	2.747	0.061	0.379
		ED	30.00 (9.83)			
		CD	40.00 (8.19)			
		CMF	28.25 (10.89)			
		EMF	29.80 (9.26)			
		FW	47.33 (6.81)			
Z4	1st half	GK	–	1.821	0.169	0.288
		ED	29.25 (21.19)			
		CD	38.67 (11.72)			
		CMF	25.00 (12.86)			
		EMF	44.60 (8.47)			
		FW	37.00 (13.23)			
	2nd half	GK	–	0.683	0.613	0.132
		ED	33.00 (5.60)			
		CD	33.67 (15.14)			
		CMF	29.13 (11.17)			
		EMF	25.00 (11.25)			
		FW	23.67 (1.53)			
Z5	1st half	GK	–	0.303	0.872	0.063
		ED	11.25 (7.68)			
		CD	21.67 (37.53)			
		CMF	23.50 (26.75)			
		EMF	27.20 (26.34)			
		FW	15.00 (6.56)			
	2nd half	GK	–	1.799	0.173	0.286
		ED	18.00 (14.54)			
		CD	1.67 (2.08)			
		CMF	11.38 (10.72)			
		EMF	10.40 (9.99)			

**Legend:** % HRmax average, average of the % heart rate max achieved during the period of analysis; Z1, % of time in Zone 1 of heart rate intensity; Z2, % of time in Zone 2 of heart rate intensity; Z3, % of time in Zone 3 of heart rate intensity; Z4, % of time in Zone 4 of heart rate intensity; and Z5, % of time in Zone 5 of heart rate intensity.

**Table 2:** One-way ANOVA values of tactical positions in each half in in % HRmax average, Z1, Z2, Z3, Z4, and Z5.

Variable	Position	Half	M(SD)	F	*p*	$\eta_{p}^{2}$
% HRmax average	GK	1st Half	–	–	–	–
		2nd Half	–			
	ED	1st Half	69.00 (15.94)	1.653	0.246	0.216
		2nd Half	79.75 (5.06)			
	CD	1st Half	82.33 (7.57)	1.213	0.333	0.233
		2nd Half	77.00 (3.61)			
	CMF	1st Half	70.63 (17.85)	0.467	0.505	0.032
		2nd Half	75.25 (6.90)			
	EMF	1st Half	85.00 (4.90) ^b^	8.679	0.019	0.520
		2nd Half	74.20 (6.57) ^a^			
	FW	1st Half	67.00 (18.52)	0.032	0.867	0.008
		2nd Half	69.00 (5.57)			
Z1	GK	1st Half	–	–	–	–
		2nd Half	–			
	ED	1st Half	7.50 (9.26)	0.325	0.589	0.051
		2nd Half	4.25 (6.65)			
	CD	1st Half	0.33 (0.58)	1.991	0.231	0.332
		2nd Half	5.33 (6.11)			
	CMF	1st Half	8.50 (12.87)	0.346	0.566	0.024
		2nd Half	12.00 (10.85)			
	EMF	1st Half	0.80 (1.79) ^b^	5.582	0.046	0.411
		2nd Half	8.60 (7.16) ^a^			
	FW	1st Half	9.67 (8.74)	0.014	0.912	0.003
		2nd Half	9.00 (4.58)			
Z2	GK	1st Half	–	–	–	–
		2nd Half	–			
	ED	1st Half	7.75 (5.68)	2.435	0.170	0.289
		2nd Half	14.75 (6.95)			
	CD	1st Half	4.00 (4.58) ^b^	18.893	0.012	0.825
		2nd Half	19.33 (4.04) ^a^			
	CMF	1st Half	6.00 (5.83) ^b^	12.548	0.003	0.473
		2nd Half	19.25 (8.83) ^a^			
	EMF	1st Half	4.40 (3.65) ^b^	29.336	0.001	0.786
		2nd Half	26.20 (8.23) ^a^			
	FW	1st Half	7.33 (5.51) ^b^	10.125	0.033	0.717
		2nd Half	19.33 (3.51) ^a^			
Z3	GK	1st Half	–	–	–	–
		2nd Half	–			
	ED	1st Half	19.25 (13.07)	1.727	0.237	0.224
		2nd Half	30.00 (9.83)			
	CD	1st Half	35.33 (26.84)	0.083	0.788	0.020
		2nd Half	40.00 (8.19)			
	CMF	1st Half	24.50 (17.91)	0.256	0.621	0.018
		2nd Half	28.25 (10.89)			
	EMF	1st Half	23.00 (14.83)	0.756	0.410	0.086
		2nd Half	29.80 (9.26)			
	FW	1st Half	31.00 (8.72)	6.542	0.063	0.621
		2nd Half	47.33 (6.81)			
Z4	GK	1st Half	–	–	–	–
		2nd Half	–			
	ED	1st Half	29.25 (21.19)	0.117	0.744	0.019
		2nd Half	33.00 (5.60)			
	CD	1st Half	38.67 (11.72)	0.205	0.675	0.049
		2nd Half	33.67 (15.14)			
	CMF	1st Half	25.00 (12.86)	0.469	0.505	0.032
		2nd Half	29.13 (11.17)			
	EMF	1st Half	44.60 (8.47) ^b^	9.686	0.014	0.548
		2nd Half	25.00 (11.25) ^a^			
	FW	1st Half	37.00 (13.23)	3.008	0.158	0.429
		2nd Half	23.67 (1.53)			
Z5	GK	1st Half	–	–	–	–
		2nd Half	–			
	ED	1st Half	11.25 (7.68)	0.674	0.443	0.101
		2nd Half	18.00 (14.54)			
	CD	1st Half	21.67 (37.53)	0.849	0.409	0.175
		2nd Half	1.67 (2.08)			
	CMF	1st Half	23.50 (26.75)	1.417	0.254	0.092
		2nd Half	11.38 (10.72)			
	EMF	1st Half	27.20 (26.34)	1.778	0.219	0.182
		2nd Half	10.40 (9.99)			
	FW	1st Half	15.00 (6.56) ^b^	13.902	0.020	0.777
		2nd Half	0.67 (1.15) ^a^			

Significantly different compared with GK ^a^; ED ^b^ at *p* < 0.05. **Legend:** % HRmax average, average of the % heart rate max achieved during the period of analysis; Z1, % of time in Zone 1 of heart rate intensity; Z2, % of time in Zone 2 of heart rate intensity; Z3, % of time in Zone 3 of heart rate intensity; Z4, % of time in Zone 4 of heart rate intensity; and Z5, % of time in Zone 5 of heart rate intensity.

**Table 3:** One-way ANOVA values of halves in each tactical position in Volume of Play, Efficiency Index, and Performance Score.

Variable	Half	Position	M(SD)	F	*p*	$\eta_{p}^{2}$
Volume of Play	1st half	GK	6.50 (2.12) ^d^	3.448	0.026	0.519
		ED	14.25 (6.29)			
		CD	18.25 (5.62)			
		CMF	24.00 (6.96) ^a^			
		EMF	17.75 (6.24)			
		FW	11.00 (1.41)			
	2nd half	GK	7.50 (5.31)	0.221	0.948	0.065
		ED	10.75 (3.75)			
		CD	11.75 (3.75)			
		CMF	13.67 (3.06)			
		EMF	11.75 (3.75)			
		FW	11.50 (5.31)			
Efficiency Index	1st half	GK	1.05 (0.96)	2.922	0.046	0.477
		ED	0.67 (0.36)			
		CD	1.01 (0.31)			
		CMF	0.71 (0.34)			
		EMF	0.24 (0.18)			
		FW	0.13 (0.09)			
	2nd half	GK	0.55 (0.63)	0.840	0.541	0.208
		ED	0.42 (0.27)			
		CD	0.69 (0.61)			
		CMF	0.48 (0.34)			
		EMF	0.20 (0.18)			
		FW	0.20 (0.15)			
Performance Score	1st half	GK	13.70 (10.70)	1.949	0.142	0.379
		ED	13.99 (6.74)			
		CD	19.25 (5.77)			
		CMF	19.10 (6.32)			
		EMF	11.30 (4.37)			
		FW	6.75 (1.18)			
	2nd half	GK	9.28 (8.09)	0.303	0.904	0.087
		ED	9.61 (6.04)			
		CD	12.79 (9.06)			
		CMF	11.59 (8.01)			
		EMF	7.83 (4.29)			
		FW	7.79 (0.41)			

Significantly different compared with GK ^a^; CMF ^d^ at *p* < 0.05. **Legend:** % HRmax average, average of the % heart rate max achieved during the period of analysis; Z1, % of time in Zone 1 of heart rate intensity; Z2, % of time in Zone 2 of heart rate intensity; Z3, % of time in Zone 3 of heart rate intensity; Z4, % of time in Zone 4 of heart rate intensity; and Z5, % of time in Zone 5 of heart rate intensity.

**Table 4:** One-way ANOVA values of tactical positions in each half in Volume of Play, Efficiency Index, and Performance Score.

Variable	Position	Half	M(SD)	F	*p*	$\eta_{p}^{2}$
Volume of Play	GK	1st Half	6.50 (2.12)	0.118	0.764	0.056
		2nd Half	7.50 (3.53)			
	ED	1st Half	14.25 (6.29)	0.530	0.494	0.081
		2nd Half	10.75 (7.27)			
	CD	1st Half	18.25 (5.62)	2.491	0.166	0.293
		2nd Half	11.75 (6.02)			
	CMF	1st Half	24.00 (6.96)	4.466	0.061	0.309
		2nd Half	13.67 (9.75)			
	EMF	1st Half	17.75 (6.24)	1.678	0.243	0.219
		2nd Half	11.75 (6.85)			
	FW	1st Half	11.00 (1.41)	0.077	0.808	0.037
		2nd Half	11.50 (2.12)			
Efficiency Index	GK	1st Half	1.05 (0.96)	0.365	0.607	0.154
		2nd Half	0.55 (0.63)			
	ED	1st Half	0.68 (0.36)	1.362	0.287	0.185
		2nd Half	0.42 (0.27)			
	CD	1st Half	1.01 (0.31)	0.871	0.387	0.127
		2nd Half	0.69 (0.61)			
	CMF	1st Half	0.71 (0.34)	1.468	0.254	0.128
		2nd Half	0.48 (0.34)			
	EMF	1st Half	0.24 (0.18)	0.139	0.722	0.023
		2nd Half	0.20 (0.18)			
	FW	1st Half	0.13 (0.09)	0.423	0.582	0.175
		2nd Half	0.20 (0.15)			
Performance Score	GK	1st Half	13.70 (10.70)	0.218	0.687	0.098
		2nd Half	9.28 (8.09)			
	ED	1st Half	13.99 (6.74)	0.938	0.370	0.135
		2nd Half	9.61 (6.04)			
	CD	1st Half	19.25 (5.80)	1.442	0.275	0.194
		2nd Half	12.79 (9.06)			
	CMF	1st Half	19.10 (6.32)	3.253	0.101	0.245
		2nd Half	11.59 (8.01)			
	EMF	1st Half	11.30 (4.37)	1.283	0.301	0.176
		2nd Half	7.83 (4.29)			
	FW	1st Half	6.75 (0.18)	10.911	0.081	0.845
		2nd Half	7.79 (0.41)			

**Legend:** % HRmax average, average of the % heart rate max achieved during the period of analysis; Z1, % of time in Zone 1 of heart rate intensity; Z2, % of time in Zone 2 of heart rate intensity; Z3, % of time in Zone 3 of heart rate intensity; Z4, % of time in Zone 4 of heart rate intensity; and Z5, % of time in Zone 5 of heart rate intensity.

**Table 5:** One-way ANOVA values of halves in each tactical position in % InDegree, % OutDegree, and % Betweenness.

Variable	Half	Position	M(SD)	F	*p*	$\eta_{p}^{2}$
%InDegree	1st half	GK	0.00 (0.00)	1.908	0.149	0.374
		ED	9.46 (4.80)			
		CD	10.18 (3.92)			
		CMF	11.81 (6.04)			
		EMF	8.80 (3.42)			
		FW	7.69 (5.90)			
	2nd half	GK	0.00 (0.00)	2.909	0.047	0.476
		ED	11.07 (7.88)			
		CD	9.95 (3.43)			
		CMF	12.98 (5.10)			
		EMF	6.92 (1.20)			
		FW	5.20 (0.09)			
% OutDegree	1st half	GK	0.00 (0.00) ^d^	2.976	0.044	0.482
		ED	7.63 (4.34)			
		CD	9.75 (4.39)			
		CMF	12.38 (3.82) ^a^			
		EMF	9.21 (4.72)			
		FW	9.46 (2.21)			
	2nd half	GK	0.00 (0.00) ^d^	6.304	0.002	0.663
		ED	6.94 (1.30) ^d^			
		CD	8.24 (2.90)			
		CMF	14.58 (5.13) ^a,b^			
		EMF	8.85 (2.92)			
		FW	8.22 (0.47)			
% Betweenness	1st half	GK	0.00 (0.00)	1.484	0.250	0.317
		ED	2.21 (1.83)			
		CD	3.46 (0.80)			
		CMF	5.39 (4.45)			
		EMF	4.39 (2.03)			
		FW	4.83 (1.42)			
	2nd half	GK	0.00 (0.00)	1.479	0.251	0.316
		ED	6.82 (10.21)			
		CD	5.45 (2.75)			
		CMF	11.73 (7.42)			
		EMF	4.73 (5.82)			
		FW	0.84 (0.39)			

Significantly different compared with GK ^a^; ED ^b^; CMF ^d^ at *p* < 0.05. **Legend:** % HRmax average, average of the % heart rate max achieved during the period of analysis; Z1, % of time in Zone 1 of heart rate intensity; Z2, % of time in Zone 2 of heart rate intensity; Z3, % of time in Zone 3 of heart rate intensity; Z4, % of time in Zone 4 of heart rate intensity; and Z5, % of time in Zone 5 of heart rate intensity.

**Table 6:** One-way ANOVA values of tactical positions in each half in % InDegree, % OutDegree, and % Betweenness.

Variable	Position	Half	M(SD)	F	*p*	$\eta_{p}^{2}$
% InDegree	GK	1st Half	0.00 (0.00)	0.001	0.999	0.001
		2nd Half	0.00 (0.00)			
	ED	1st Half	9.46 (4.80)	0.121	0.740	0.020
		2nd Half	11.07 (7.88)			
	CD	1st Half	10.18 (3.92)	0.008	0.932	0.001
		2nd Half	9.95 (3.43)			
	CMF	1st Half	11.81 (6.04)	0.131	0.725	0.013
		2nd Half	12.98 (5.10)			
	EMF	1st Half	8.80 (3.42)	1.076	0.340	0.152
		2nd Half	6.92 (1.20)			
	FW	1st Half	7.69 (5.90)	0.356	0.611	0.151
		2nd Half	5.20 (0.09)			
% OutDegree	GK	1st Half	0.00 (0.00)	0.001	0.999	0.001
		2nd Half	0.00 (0.00)			
	ED	1st Half	7.63 (4.34)	0.094	0.770	0.015
		2nd Half	6.94 (1.30)			
	CD	1st Half	9.75 (4.39)	0.331	0.586	0.052
		2nd Half	8.24 (2.90)			
	CMF	1st Half	12.38 (3.82)	0.709	0.420	0.066
		2nd Half	14.58 (5.13)			
	EMF	1st Half	9.21 (4.72)	0.018	0.899	0.003
		2nd Half	8.85 (2.92)			
	FW	1st Half	9.46 (2.21)	0.596	0.521	0.230
		2nd Half	8.22 (0.47)			
% Betweenness	GK	1st Half	0.00 (0.00)	0.001	0.999	0.001
		2nd Half	0.00 (0.00)			
	ED	1st Half	2.21 (1.83)	0.790	0.408	0.116
		2nd Half	6.82 (10.21)			
	CD	1st Half	3.46 (0.80)	1.938	0.213	0.244
		2nd Half	5.45 (2.75)			
	CMF	1st Half	5.39 (4.45)	3.214	0.103	0.243
		2nd Half	11.73 (7.42)			
	EMF	1st Half	4.39 (2.03)	0.012	0.916	0.002
		2nd Half	4.73 (5.82)			
	FW	1st Half	4.83 (1.42)	14.664	0.062	0.880
		2nd Half	0.84 (0.39)			

**Legend**: % HRmax average, average of the % heart rate max achieved during the period of analysis; Z1, % of time in Zone 1 of heart rate intensity; Z2, % of time in Zone 2 of heart rate intensity; Z3, % of time in Zone 3 of heart rate intensity; Z4, % of time in Zone 4 of heart rate intensity; and Z5, % of time in Zone 5 of heart rate intensity.

## Abbreviations

The following abbreviations are used in this manuscript: Heart Rate (HR), Maximum Heart rate (HRmax).
